# Attitudes towards using electronic health records of patients with psoriasis and dermatologists: a cross-sectional study

**DOI:** 10.1186/s12911-020-01302-y

**Published:** 2020-12-30

**Authors:** Toni Maria Klein, Matthias Augustin, Natalia Kirsten, Marina Otten

**Affiliations:** Institute for Health Services Research in Dermatology and Nursing (IVDP), University Medical Center Hamburg-Eppendorf (UKE), Cvderm, Martinistraße 52, 20246 Hamburg, Germany

**Keywords:** Electronic health record, EHR, Dermatology, Psoriasis, Patients, Physicians, Attitudes

## Abstract

**Background:**

Electronic health records (EHRs) offer various advantages for healthcare delivery, especially for chronic and complex diseases such as psoriasis. However, both patients’ and physicians’ acceptability is required for EHRs to unfold their full potential. Therefore, this study compares patients’ and physicians’ attitudes towards using EHRs in routine psoriasis care.

**Methods:**

For the purpose of this study, a questionnaire was developed based on literature research and analyses of previously conducted focus groups. Participants completed either a paper-based or an electronic version of the questionnaire. Patient recruitment took place at an dermatological outpatient clinic and via several online pathways (patient associations, and social media). Physicians were recruited via a mailing list of a dermatological association and at a dermatological conference. Patients’ and physicians’ responses were compared using *χ*^2^ tests and Fisher’s exact tests.

**Results:**

The study consisted of 187 patients and 44 dermatologists. Patients compared to physicians rated almost all potential EHR uses as significantly more important and expected significantly more potential benefits from EHRs.

**Conclusions:**

Patients showed positive expectations towards using EHRs, whereas there was more scepticism in the physician sample. This aligns with previous findings. These differences illustrate the necessity to involve all stakeholders, especially patients and physicians, into the process of developing and implementing EHRs.

## Background

Digitalisation entails a variety of innovations for the healthcare system, which are supposed to support the provision of healthcare, to foster the interaction between different stakeholders, and to further empower patients [1]. For most industrialised countries it is vital to make use of these developments in order to reduce healthcare costs while sustaining or even increasing treatment quality [2]. In the course of this development, electronic health records (EHRs) are gaining importance. The innovative character of EHRs can widely differ as their scope can range from a folder for collecting scanned documents up to an application, in which data are being entered and stored, and which provides automated analyses and alerts [3]. Well-developed EHRs are supposed to reduce administrative burden, improve flow of information, and ease therapy management [3, 4].

Implementing EHRs is a multi-stakeholder process and hence human and socio-organisational factors need to be considered throughout development [5]. Focusing on patients and physicians is crucial as they are major decision makers and their attitudes might have greater impact on the usage of EHRs than the attitudes of nursing or assistant staff who have less autonomy in their work [6].

Depending on the design of an EHR and its field of application, the involvement of these stakeholders into the process of data management might vary. Especially for patients, the degree of participation can range from having no access to data, over accessing data that professionals already entered until entering data on their own [4]. EHRs with patient engagement in data management have great potential to improve treatment processes by increasing overall patient involvement [7] and by supplementing clinical data with patient-reported outcomes [8]. Patient-reported outcomes are gaining importance in increasing patient-centred healthcare systems, which are characterised by valuing patients as individual persons and emphasising the relationship between patients and care providers [9]. Including the patient perspective into the treatment process should automatically induce joint discussions between patients and physicians leading to a mutually reached treatment plan as pronounced in the concept of shared decision-making (SDM) [10]. EHRs have the potential to anchor SDM into consultations and hence to contribute largely to the healthcare delivery process, especially for patients with complex and chronic conditions who need frequent and comprehensive care. Therefore, one promising area for implementing EHRs is psoriasis care. Psoriasis is a chronic condition with multiple triggering factors [11], which is often burdensome for patients [12], associated with various comorbidities [11], and poses high economic burden for individuals and society at large [11, 13].

A precondition for usage of innovations is users’ acceptability [14]. Recent literature on the acceptability of EHRs mostly focusses on one specific stakeholder group but rarely addresses several perspectives simultaneously. Results from recent studies reveal that patients endorse to take a more active role in therapy management and gain more control [15, 16]. Additionally, they feel that EHRs can improve communication between patients and physicians such as between different healthcare providers [17]. Nevertheless, patients express the necessity of physicians’ awareness, acceptance, and use in order to achieve comprehensive and hence successful implementation [17]. In contrast, physicians are rather concerned that using EHRs might decrease efficiency [18] and that maintenance of data would cut time for face-to-face care [19]. A Brazilian study investigating user satisfaction with EHRs among both patients and physicians reveals that almost all patients valued the use of EHRs at the point of care, whereas physicians’ initial endorsement of the EHR decreased after implementation [20].

These results suggest that attitudes of patients and physicians vary and that both interest groups approach the implementation of EHRs with different expectations. However, no study has been identified which directly compares patients’ and physicians’ attitude. Additionally, studies are scarce investigating acceptability of either patients with or physicians treating dermatologic diseases.

Therefore, the aim of this study was to investigate and compare the attitude towards EHRs from the perspective of both patients with psoriasis and dermatologists treating this skin disease.

## Methods

In this observational cross-sectional study, patients and physicians were recruited. Inclusion criteria for patients were being German speaking, being able to read and answer questionnaires, giving informed consent, and having psoriasis. No specific exclusion criteria were formulated to recruit a diverse sample and to minimise selection bias. Inclusion criteria for physicians were being dermatologists and treating patients with psoriasis, whereas no specific exclusion criteria were formulated. Recruitment took place online and offline via several pathways. Patients were recruited:

- In person at an outpatient dermatology care unit of the University Medical Center Hamburg-Eppendorf using a paper-based questionnaire.
- Online via Facebook in a German private group from and for patients with psoriasis encompassing 9000 members at the time of recruitment. Into this group, a call for participation was posted including the link to the electronic questionnaire.
- Online via websites of two German psoriasis associations (Psoriasis-Netz, Deutscher Psoriasis Bund e.V.), where a call for participation and the link to the electronic questionnaire was provided.
- Online via e-mail, which was sent by one German psoriasis association (Deutscher Psoriasis Bund e.V.) to 300 randomly selected members including a call for participation and the link to the electronic questionnaire.

Dermatologists were recruited:

- In person at a dermatological conference (Fortbildungswoche für praktische Dermatologie und Venerologie) using a paper-based questionnaire.
- Online via e-mail, which was sent by a German dermatologists’ association (Berufsverband der Deutschen Dermatologen) to 200 randomly selected members.

Targeted sample size was 50 participants per group to allow for regression analyses [21]; however, for the present manuscript, only descriptive and bivariate statistics were required. Data collection lasted from July to August 2018.

Development of the questionnaire was based on literature research and on results from previously conducted focus groups with patients with psoriasis (described in more detail in [22]). Patient questions were developed at first and later adapted for the physician questionnaire accordingly (e.g. patient: “Communication with my physician can improve.”; physician: “Communication with my patients can improve.”). The present article analyses items on participants’ attitudes towards EHRs and towards visualisation of outcomes, specifically asking for the importance of potential EHR uses (“How important are the following statements to you?”) and expectation of potential EHR benefits (“To what extent do you agree with the following statements?”/“How realistic do you think the following statements are?”). Analyses include items that were posed to patients and physicians as well as items that were exclusively posed to one group. The questionnaires encompassed further items, which are not part of this article and will be published elsewhere [22].

Participants answered items on five-point Likert scales. For analysis, Likert scales were condensed into three categories, presenting one positive [“(very) important”, “(very) realistic”, “(totally) agree”], one neutral (“neither nor”), and one negative answer [“(very) unimportant”, “(very) unrealistic”, “(totally) disagree”]. Cases with missing values were excluded from analysis if missing values did not exceed 5%. Participants’ characteristics were analysed using descriptive statistics, chi-squared test (*χ*^2^ test), and independent t-test. Comparison of response behaviours were conducted using cross tables, *χ*^2^ test, and Fisher’s exact test (FET) if cross tables had more than 20% of cells with expected counts below five. A significance level of α = 0.05 was applied. For investigating the direction of association, adjusted standardised residuals were calculated with values below − 1.96 and above 1.96 revealing significance. Only participants answering at least one relevant question were included in the analysis.

## Results

In total, 190 patients returned questionnaires, of which three were excluded due to unanswered items on topics about EHRs, resulting in 187 patient questionnaires. Of these, 72.7% (n = 136) completed the electronic and 27.3% (n = 51) completed the paper-based questionnaire. The patient sample encompassed 51.4% (n = 93) men and revealed a mean age of 51.62 years [standard deviation (SD) = 15.26; median (mdn) = 55.00; minimum (min) = 15.00; maximum (max) = 93.00].

The physician sample encompassed 44 participants, of which 86.4% (n = 38) completed the electronic and 13.6% (n = 6) completed the paper-based questionnaire. The physician sample consisted of 68.2% (n = 30) men and revealed a mean age of 53.30 years (SD = 7.95, mdn = 56.00; min = 37.00; max = 67.00). The items revealed a maximum number of missing values of n = 1 (2.27%) in the physicians’ sample and n = 4 (2.15%) in the patients’ sample, which is below the threshold of 5%. Accordingly, participants with missing values were excluded from the analysis of the respective item.

Both samples differed significantly with regard to distribution of gender [*χ*^2^ (1, n = 225) = 4.031, *p* = 0.045], but not with regard to age [t (128.589) = − 1.020, *p* = 0.310] and mode of questionnaire administration [*χ*^2^ (1, n = 231) = 3.564, *p* = 0.059].

Both patients and physicians thought it would be important that physicians can retrieve data quickly and easily due to the use of an EHR (patients: 95.1% vs. physicians: 88.6%; *p* = 0.181, FET). However, patients’ and physicians’ responses differed significantly with regard to the five other items about the importance of potential EHR uses: Patients rated items that ask for patients’ access to their own data (93.4% vs. 48.8%; *p* < 0.001, FET) and for patients’ access to visualised data (86.8% vs. 63.6%; *p* = 0.001, FET) more often as (very) important. The same accounts for the possibility for physicians to look at visualised data (92.3% vs. 72.7%; *p* = 0.001, FET). Moreover, patients rated discussion between patients and physicians based on visualised data (90.7% vs. 70.5%; *p* = 0.002, FET) as well as communication and cooperation between all stakeholders involved (94.0% vs. 81.8%; *p* = 0.027, FET) more often as (very) important.

**Fig. 1:**
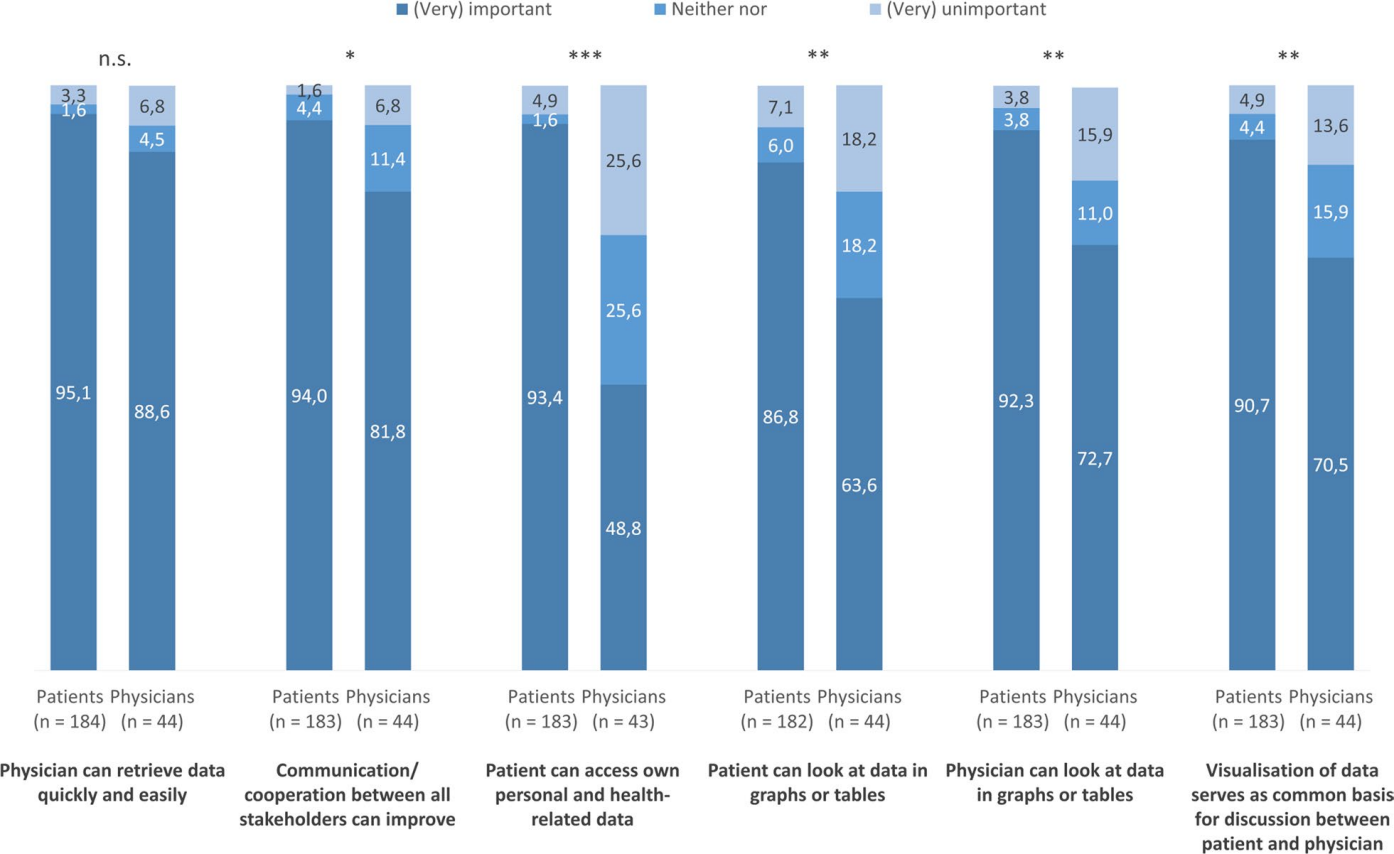
Importance of potential electronic health records (EHR) uses from patients’ and physicians’ perspective from (very) important to (very) unimportant. Significance of differences between patients and physicians (*χ*^2^ and Fisher’s exact test); *p < 0.05, **p < 0.01, ***p < 0.001; n.s., not significant

**Fig. 2:**
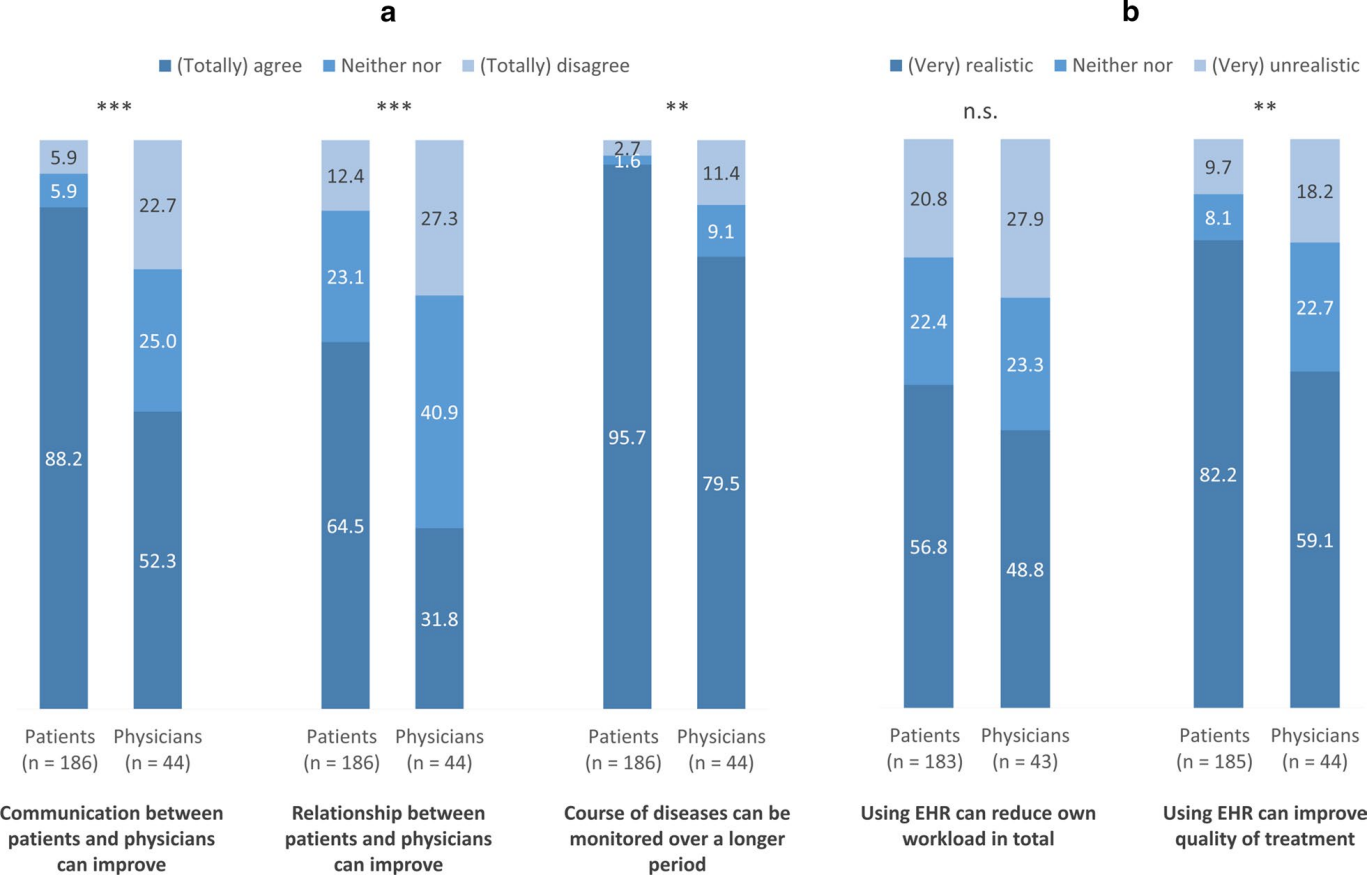
Expectations of potential electronic health records (EHR) benefits from patients’ and physicians’ perspective from (totally) agree to (totally) disagree (**a**) and (very) realistic to (very) unrealistic (**b**). Significance of differences between patients and physicians (*χ*^2^ and Fisher’s exact test); *p < 0.05, **p < 0.01, ***p < 0.001; n.s., not significant

Similar to the importance of potential EHR uses (Fig. 1), patients more often expected benefits due to the implementation of EHRs in clinical routine (Fig. 2). Patients (totally) agreed more often (95.7% vs. 79.5%; *p* = 0.002, FET) that by using an EHR, the course of the disease can be monitored longitudinally. Great differences are observable with regard to the expectations that by using an EHR, the communication (88.2% vs. 52.3%; *p* < 0.001, FET) and the relationship [64.5% vs. 40.9%; *χ*^2^ (2, n = 230) = 15.972, *p* < 0.001] between patients and physicians can improve. Within both samples, only around half of the participants expected reduced workload due to the use of an EHR [56.8% vs. 48.8%; *χ*^2^ (2, n = 226) = 1.216, *p* = 0.544]. However, patients expected more often that the use of an EHR can improve quality of the treatment (82.2% vs. 59.1%; *p* = 0.003, FET).

**Fig. 3:**
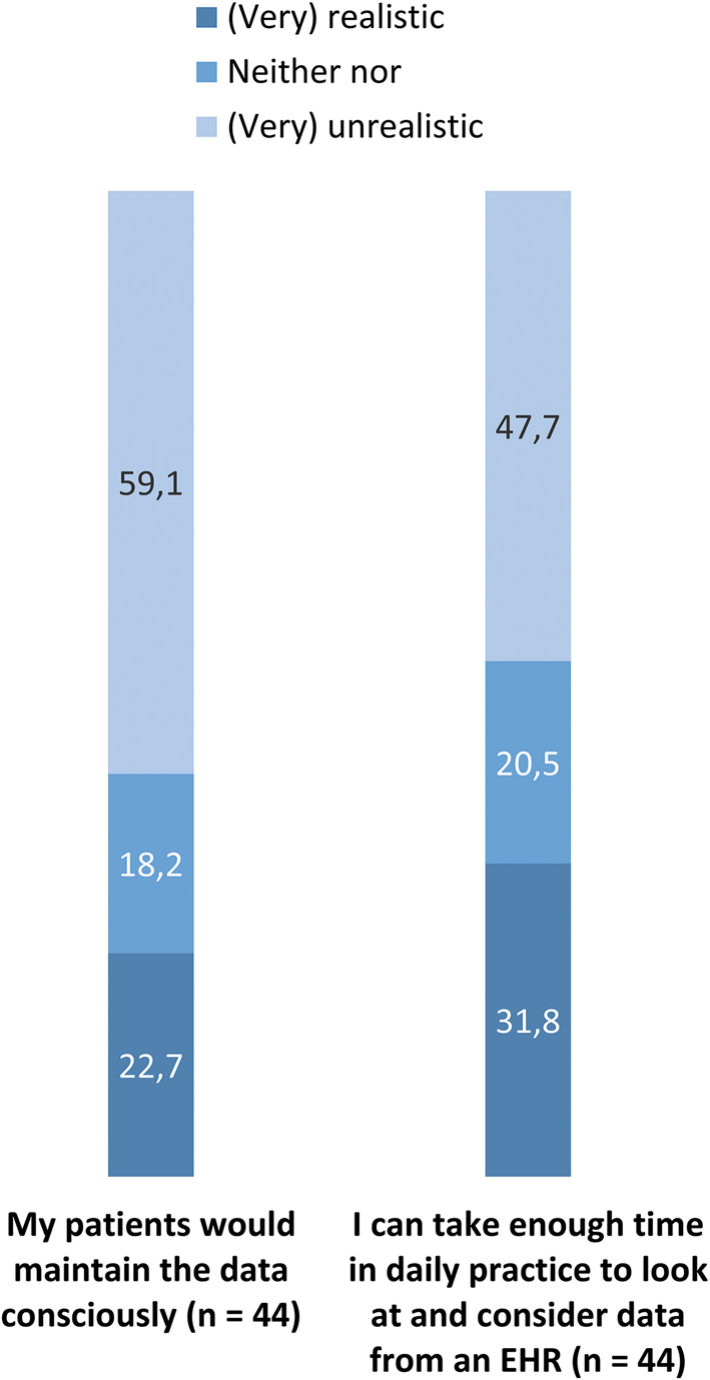
Physician-specific questions on expectations towards implementation of electronic health records

With regard to the physician-specific questions (Fig. 3), less than one-third (n = 14, 31.8%) of the sample regarded it as (very) realistic to have enough time in daily practice to look at and consider data from an EHR. Moreover, less than one-fourth (n = 10, 22.7%) thought that their patients would maintain EHR data consciously. Respectively, 47.7% (n = 21) and 59.1% (n = 26) of the physicians regarded both items as (very) unrealistic.

**Fig. 4:**
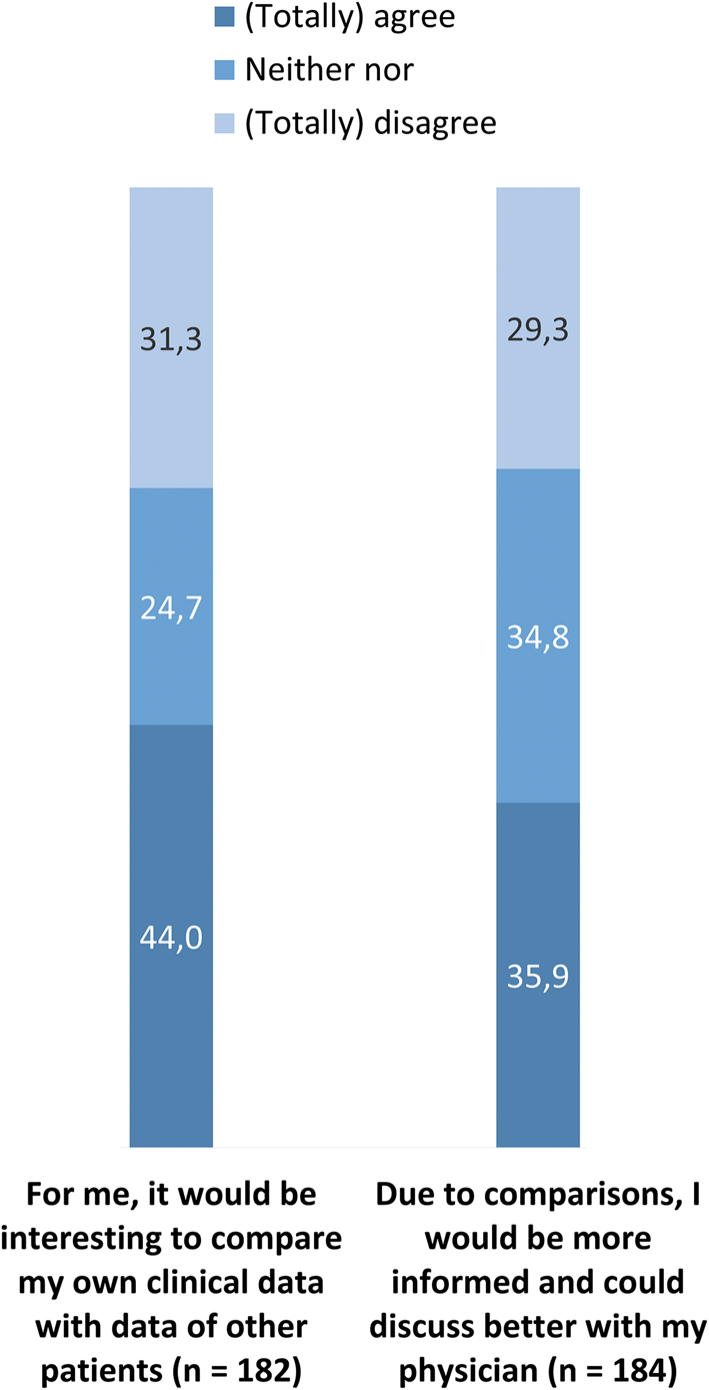
Patient-specific questions on inter-patient comparison with data in electronic health records

With regards to patient-specific questions (Fig. 4), less than half (n = 80, 44.0%) of the patients would find it interesting to compare their own data with data of other patients. Only 35.9% (n = 66) thought that they could discuss more informed with their physicians when comparing their data with data of other patients. Respectively, 24.7% (n = 45) and 34.8% (n = 64) of the patients were undecided about these items.

## Discussion

The aim of this study was to assess the acceptability of EHRs in patients with psoriasis and dermatologists treating this disease and to compare the results of both groups. The results reveal that patients with psoriasis regard a variety of potential EHR uses as important and that they expect overall improvements. In contrast, physicians express higher scepticism with regard to the implementation of EHRs. Differences between both samples are noticeable for a variety of potential uses and benefits, but especially pronounced with regard to the possibility for patients to access data.

The findings in this study align with previous results. Studies about patients’ attitudes from the UK [16], USA [7], Germany [15], and Australia [17] also revealed that patients largely expected or experienced improvements in patient involvement and increased treatment quality due to the use of EHRs. Physicians in a Turkish study expressed that they expected several benefits from using an EHR but only few expected benefits with regard to doctor-patient communication, communication between healthcare providers, or in reduction of medical errors [23]. Grabenbauer et al. [19] report that American physicians were concerned that data usage reduces time for direct patient care and Hackl et al. [24] state that Austrian physicians doubted benefits but feared additional workload due to the implementation of EHRs. Overall, both previous and our results reveal that patients largely expect and partly experienced benefits due to using EHRs. In contrast, physicians show more scepticism with regard to the implementation and a variety of potential outcomes of using EHRs.

This study investigated patients’ and physicians’ attitudes and expectancies towards the implementation and routine use of EHRs. Focusing on patients and physicians derived from separate findings within both groups suggesting that considerable differences are conjecturable. The results of the present study confirm this assumption. The significant differences between both groups as well as the necessity that both patients and physicians actively engage in the use of EHRs [7, 17] support the approach of specifically concentrating on patients and physicians. With respect to the significant lower levels of expectation towards EHRs on the side of physicians it is of great concern to increase the acceptability in physicians. In a Brazilian study, physicians’ endorsement for an EHR decreased after its implementation. However, a Turkish study reveals that the majority of physicians and other healthcare professionals report at least some improvements in their daily routine due to the use of an EHR. Similarly, several studies in patient samples reveal that frequent use of an application lead to greater acceptability [25–27]. Hence, enabling physicians to experience benefits of EHRs and to adapt to new requirements and tasks might also increase physicians’ acceptability and finally increase benefits due to EHR use. Simultaneously, physicians need to recognise the patients’ autonomy and willingness to be involved in the treatment process.

Analysing attitudes and expectancies in this study derived from findings, which reveal that users’ expectations and perceived usefulness of innovations are important indicators for their acceptance and adoption [28, 29]. However, differences between patients and physicians found in this study pronounce the necessity to involve all stakeholders early in the development and implementation process to facilitate great usability and therefore usage of EHRs. Previous studies show that the perspective of providers significantly differs from this of vendors [30] and that physicians who were not included in the implementation process are likely to have a sceptical attitude, feel poorly informed, feel other-directed, and express concerns about additional workload and data privacy [24].

A limitation of the study is that results cannot be generalised due to the sampling strategy. The large share of participants completing the electronic version might indicate a higher number of technology-affine patients and physicians than it could be expected on average. Additionally, recruiting in the outpatient clinic might have resulted in a sample of patients with relatively severe forms of psoriasis. However, considering the attitude of these patients when implementing EHRs might be especially promising as those might be the ones with the most complex courses of psoriasis requiring comprehensive therapy management [31, 32]. Notably, for such complex therapy courses, EHRs may improve patient safety due to reduced medication errors and provided clinical guidance [33]. Especially, when EHR implementation is accompanied by larger strategies, this can increase patient safety directly and indirectly. However, it remains questionable whether such advantages will become apparent when EHRs are implemented outside of wider strategies [34]. When interpreting results of comparisons between patients and physicians, discrepancies in sample sizes should be considered. Moreover, this article disregards aspects of data privacy, which is a frequently expressed issue [2, 35]. Results on data privacy from this study were excluded from this article but have been published elsewhere [22]. These results [22] underline that patients demand to know where and how data are stored as well as who has access to it. Nevertheless, other studies indicate that concerns about data privacy might decrease when frequently using EHRs [25] and that patients’ enthusiasm might outweigh their concerns [36]. However, it is pivotal that EHRs are developed applying highest security standards to protect patient data as requested by regulators [2]. Additionally, EHRs need to be carefully implemented in a comprehensive approach including all stakeholders to reduce barriers and possible pitfalls [22].

Nevertheless, a strength of this study is that, to the best of our knowledge, this is the first study directly comparing the attitude of patients and physicians towards using EHRs in clinical routine. Additionally, no previous study could be identified investigating patients with or physicians treating psoriasis. Another strength is the diverse sample due to multiple sampling strategies giving a broad insight into the population. Furthermore, patients’ opinion was considered from beginning on by basing the questionnaire on results of focus groups. Despite the lack of additional focus groups with dermatologists, a variety of aspects mentioned by patients was transferable to the physician questionnaire. Wording of questions for both samples were kept as similar as possible.

## Conclusion

Following the results of this study, it can be strongly recommended to involve all stakeholders comprehensively in the whole implementation process of EHRs. This accounts especially for physicians and patients, who are major decision makers in psoriasis care. A low threshold to the engagement process is necessary specifically for physicians as the results suggest that involving patients might be an easier task than involving physicians. Additionally, the final application should be intuitive and easy to handle in data entry and retrieval to allow users to make positive experiences and subsequently to gain confidence in using EHRs.

## Data Availability

The datasets used and/or analysed during the current study are available from the corresponding author on reasonable request.

## Abbreviations

EHR: Electronic health record
FET: Fisher’s exact test
max: Maximum
mdn: Median
min: Minimum
SD: Standard deviation
SDM: Shared decision-making
*χ*^2^ test: Chi-squared test
